# Simple Fecal Flotation Is a Superior Alternative to Guadruple Kato Katz Smear Examination for the Detection of Hookworm Eggs in Human Stool

**DOI:** 10.1371/journal.pntd.0003313

**Published:** 2014-12-18

**Authors:** Tawin Inpankaew, Fabian Schär, Virak Khieu, Sinuon Muth, Anders Dalsgaard, Hanspeter Marti, Rebecca J. Traub, Peter Odermatt

**Affiliations:** 1 Department of Veterinary Disease Biology, Faculty of Health and Medical Science, University of Copenhagen, Copenhagen, Denmark; 2 Department of Parasitology, Faculty of Veterinary Medicine, Kasetsart University, Bangkok, Thailand; 3 Department of Epidemiology and Public Health, Swiss Tropical and Public Health Institute, Basel, Switzerland; 4 University of Basel, Basel, Switzerland; 5 National Center for Parasitology, Entomology and Malaria Control, Phnom Penh, Cambodia; 6 Medical and Diagnostics Department, Swiss Tropical and Public Health Institute, Basel, Switzerland; 7 Faculty of Veterinary and Agricultural Sciences, The University of Melbourne, Parkville, Victoria, Australia; School of Population Health, University of Queensland, Australia

## Abstract

**Background:**

Microscopy-based identification of eggs in stool offers simple, reliable and economical options for assessing the prevalence and intensity of hookworm infections, and for monitoring the success of helminth control programs. This study was conducted to evaluate and compare the diagnostic parameters of the Kato-Katz (KK) and simple sodium nitrate flotation technique (SNF) in terms of detection and quantification of hookworm eggs, with PCR as an additional reference test in stool, collected as part of a baseline cross-sectional study in Cambodia.

**Methods/Principle Findings:**

Fecal samples collected from 205 people in Dong village, Rovieng district, Preah Vihear province, Cambodia were subjected to KK, SNF and PCR for the detection (and in case of microscopy-based methods, quantification) of hookworm eggs in stool. The prevalence of hookworm detected using a combination of three techniques (gold standard) was 61.0%. PCR displayed a highest sensitivity for hookworm detection (92.0%) followed by SNF (44.0%) and quadruple KK smears (36.0%) compared to the gold standard. The overall eggs per gram feces from SNF tended to be higher than for quadruple KK and the SNF proved superior for detecting low egg burdens.

**Conclusion/Significance:**

As a reference, PCR demonstrated the higher sensitivity compared to SNF and the quadruple KK method for detection of hookworm in human stool. For microscopic-based quantification, a single SNF proved superior to the quadruple KK for the detection of hookworm eggs in stool, in particular for low egg burdens. In addition, the SNF is cost-effective and easily accessible in resource poor countries.

## Author Summary

Hookworm infection is widespread in resource-poor countries worldwide. Detection of hookworm eggs in human feces can be done by the Kato Katz technique (KK), sodium nitrate flotation technique (SNF) or PCR. This study was conducted to evaluate and compare the diagnostic parameters of the KK and simple SNF in terms of detection and quantification of hookworm eggs, with PCR as an additional reference test in stool, collected as part of a baseline cross-sectional study in Cambodia. PCR demonstrated the highest sensitivity for hookworm detection. By microscopy, SNF of a single stool sample proved superior for the detection of hookworm eggs in feces than quadruple Kato Katz smears. Hookworm egg counts were higher by SNF than those obtained using Kato Katz. Thus, the SNF proved superior to the quadruple Kato-Katz smears for the detection of low egg burdens and for the quantification of egg intensities. We propose the simple SNF is a superior alternative to the Kato-Katz for detection and quantification of hookworm infection in resource poor counties. The test is cost-effective and easily accessible.

## Introduction

Human hookworms are estimated to infect between 576–740 million people globally and are responsible for a global burden of 3.2 million disability-adjusted life years [1], [2]. Hookworms are a leading cause of iron deficiency anemia and protein malnutrition, especially among pre- and school-aged children and untreated infections are known to result in adverse maternal-fetal outcomes in pregnant women [3]. The principal intervention strategy for hookworm infection is periodic mass drug administration of humans with the benzimidazole drugs, albendazole or mebendazole.

Diagnosis of soil-transmitted helminth (STH) infections, including hookworm has largely relied on copromicroscopy techniques based on the detection and quantification of eggs in feces. These tests aim to offer simple, reliable and economical options for assessing the prevalence and intensity of STH infections and monitoring the success of drug efficacy trials and helminth control programs. Of these, the Kato-Katz (KK) technique is currently the most widely used and accepted diagnostic technique recommended by the World Health Organization (WHO) [4]. The KK technique is relatively simple, reproducible, requires minimal equipment and the kit is mostly reusable. Hence the technique is inexpensive and commonly used as a field-based or point-of-care diagnostic test. The major disadvantage of the KK technique, however, is its lack of sensitivity for the detection of low levels and low intensities of STH infections [5]. In addition, hookworm eggs rapidly disappear in cleared slides, resulting in false negative test results if the interval between preparation and examination of the slides is too long (>30 min) [6]. For these reasons, it is necessary to increase the sensitivity of the KK technique by examining single fecal samples using multiple KK smears and/or by examining multiple fecal samples over multiple consecutive days [7], [8].

The sodium nitrate flotation (SNF) technique has been used in the veterinary field for diagnosing helminth infections for the last four decades. This method is currently the diagnostic test of choice for enteric parasites in small animals (e.g. dogs, cats) and commonly utilized with a commercial reusable stand-up fecal flotation device known as the Fecalyzer (EVSCO 014008-50, USA). Recent studies suggest that fecal flotation techniques hold promise for the diagnosis of STH infections in humans. A single fecal flotation using the FLOTAC and more recently the mini-FLOTAC device has consistently been shown superior in terms of sensitivity compared to triplicate KK and ether concentration methods for the detection of hookworm eggs in stool [5], [9], albeit at the expense of lower egg counts [10], [11], [12], [13].

A number of studies have reported the superior diagnostic parameters of molecular-based diagnostic techniques compared to those of microscopy for the detection of parasite stages in feces, including hookworms [14], [15], [16], [17].We therefore utilized a previously validated polymerase chain reaction (PCR) targeting the internal transcribed spacer (ITS)-1, 5.8S and ITS-2 region of hookworms as an additional diagnostic test for assessing the sensitivity of the coproscopy-based methods. This study was conducted to evaluate and compare the diagnostic parameters of the KK and the SNF methods in terms of detection and quantification of hookworm eggs in stool collected as part of a baseline cross-sectional study in Cambodia, with PCR as an additional reference test,.

## Materials and Methods

### Ethical considerations

The research was approved by the Ethics Committee of the Cantons of Basel-Stadt and Baselland (EKBB, #18/12, dated 23 February 2012), Switzerland, and by the National Ethics Committee for Health Research, Ministry of Health, Cambodia (NECHR, #192, dated 19 November 2011). Written informed consent was obtained from each participant prior to the start of the study. For participants between the ages of 2 and 18 years, written informed consent was obtained from the parents, legal guardian or appropriate literate substitute. All participants were informed of the study's purpose and procedures prior to enrolment. All parasitic infections diagnosed were treated according to the guidelines of the National Helminth Control Program of Cambodia [18].

### Study area and field procedures

The study was conducted in Dong village, Rovieng district, Preah Vihear province, Cambodia [9]. In brief, a total of 205 persons were randomly chosen for inclusion in this cross-sectional study. Two fecal samples were collected from each enrolled participant over two consecutive days. On the day of the first visit, informed consent was obtained from all household members and questionnaire interviews were conducted [9]. To all enrolled participants, pre-labeled stool containers were distributed. Participants were asked to defecate during the morning on the following day where stool samples were collected and a second stool container distributed. One half of the collected stool samples were transported at ambient temperature to the laboratory in the Rovieng Health Center within three hours after defecation. One part (approximately 2 g) was placed into a 15 ml centrifuge tube containing 8 ml of 10% formalin for examination using SNF and the other part (approximately 2 g) was placed into a 15 ml centrifuge tube containing 8 ml of 2.5% potassium dichromate for PCR analysis and transported to the School of Veterinary Science, University of Queensland, Gatton campus, Australia. The same collection procedure of fecal samples was carried out in the morning of the second day with samples immediately subjected to a second round of examination by the KK method.

### Kato Katz technique procedure

For each stool sample two KK smears (duplicate slides) were prepared. For each person four KK smears were examined (two smears on each of two stool samples). The preparation of each slide was done following the protocol previously described [19]. Number 120-sized nylon mesh screen was used for filtering the stool and a standard plastic KK template was used to deliver 41.7 mg of stool from each sample onto each slide. The smear was examined under light microscope after allowing for clearance for 30 min. Total number of hookworm eggs observed on the slide was counted and noted. Egg counts were multiplied by 24 to obtain the number of eggs per gram feces (epg).

### Sodium nitrate flotation technique procedure

SNF was carried out according to a previously described protocol [20] on a single stool sample per study participant. Briefly, the formalin fixative was poured off and a fecal suspension prepared by thoroughly mixing approximately two gram of each stool sample with four times its volume of distilled water. The suspension was strained though a small funnel lined with two layers of surgical gauze directly into a 10 ml centrifuge tube and centrifuged for two min at 3,000× g. The supernatant was poured off leaving behind the fecal pellet (250 mg). Two mL of sodium nitrate solution (specific gravity 1.20) was added and the pellet mixed into a slurry using a wooden spatula. Sodium nitrate solution (specific gravity 1.20, or 315 gm/L of water) was then filled to the rim of the centrifuge tube, forming a positive meniscus and a 22 mm×22 mm cover slip was carefully placed on top. After 10 min, the cover slip was removed and placed onto a microscope slide. The entire slide was examined under light microscope at 100× magnification in a zig-zag fashion and the total number of hookworm eggs on the coverslip was counted. The observed number was multiplied by four to obtain the epg.

### Polymerase chain reaction procedure

Genomic DNA was extracted directly from human fecal samples using the PowerSoil DNA Kit (Mo Bio, CA, USA) according to manufacturer's instructions with minor modifications and PCR carried out as previously described [17]. A positive control of each hookworm species and a negative control of distilled water were included in each run. The PCR products were visualized on 1% agarose gels in Sodium Borate (SB) buffer and stained by SYBR safe® Nucleic Acid Gel Stain (Life Technologies, Invitrogen, Eugene, USA).

### Statistical analysis

The results of the fecal examinations were entered in EXCEL (Microsoft, USA) and analyzed by using STATA version 12.1 (StataCorp LP; College Station, TX). To estimate sensitivity, specificity and negative predictive value (NPV), results for the three techniques were categorized into positive and negative variables, presented in cross-tabulations, and compared for equal possibilities of being positive by using McNemar's test with 95% confidence interval (CI). The combination of the three techniques was used as diagnostic “gold standard” to estimate the sensitivity and specificity of each technique. Agreement among infection intensities of the two techniques (only KK and SNF) was estimated from their mean epg values, using paired student *t*-test. The “true prevalence” was calculated with the model developed by Marti and Koella, described elsewhere [21].

## Results

### Prevalence

**Figure 1:**
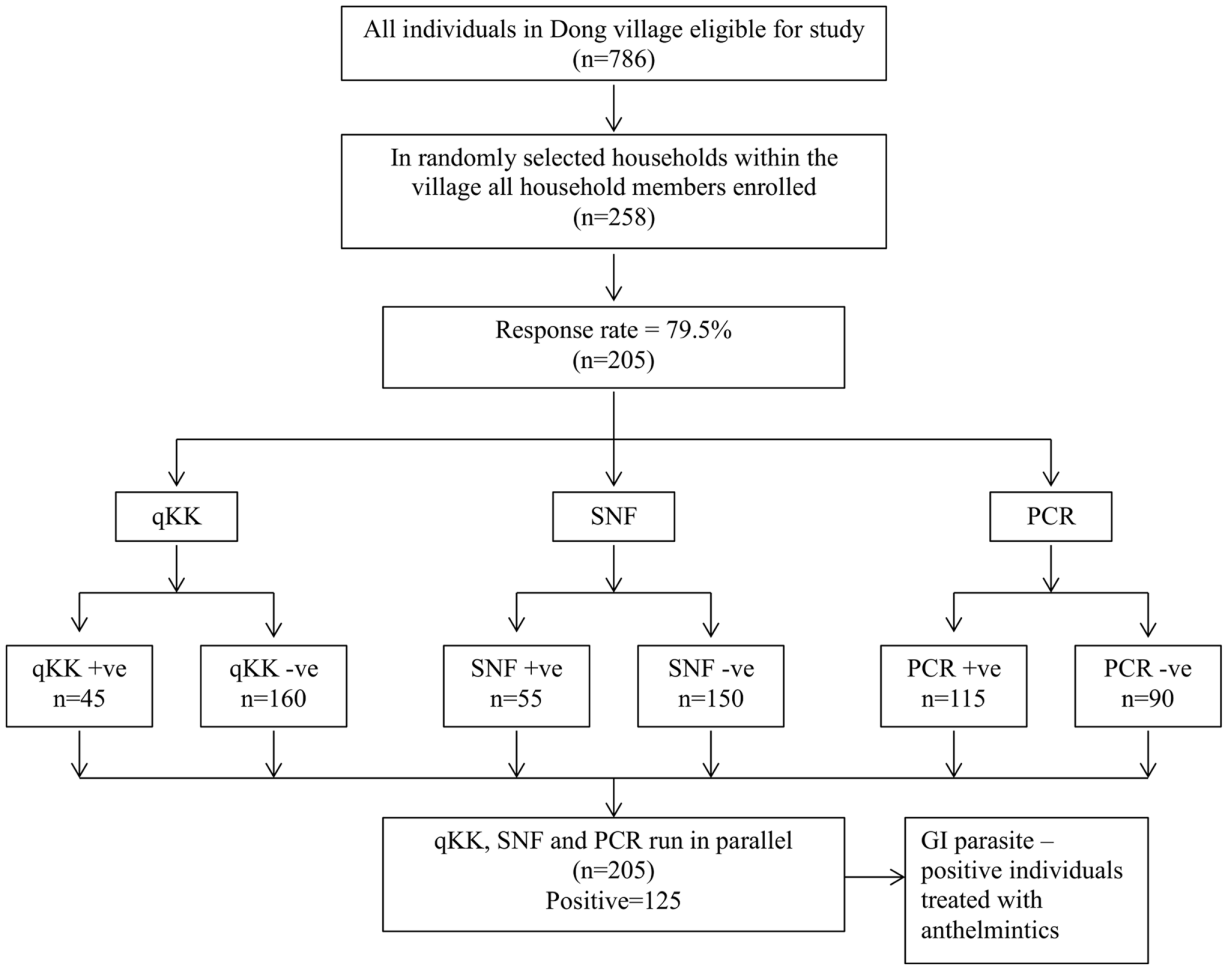
A flow diagram illustrating the study design and summary of diagnostic test results.

For a diagrammatic guide to the study design and summary of the diagnostic results refer to Fig. 1. The overall prevalence of hookworm infection in humans was 61.0% by the combined techniques, 56.1% by PCR, 26.8% by SNF and 22.0% by quadruple KK (16.6% by day 1 KK, 12.2% by day 2 KK, Table 1).

**Table 1:** Prevalence of hookworm infection, sensitivity, specificity and negative predictive value using different diagnostic techniques.

Technique	Number positive (%)	Sensitivity (%) (95% CI)	Specificity (%) (95% CI)	Negative predictive value (%) (95% CI)
KK-SNF-PCR (gold standard)	125 (61.0)	100	100	100
KK day 1	34 (16.6)	27.2 (19.6–35.9)	100	46.8 (39.1–54.6)
KK day 2	25 (12.2)	20.0 (13.4–28.1)	100	44.4 (37.1–52.0)
KK day 1+day 2	45 (22.0)	36.0 (27.6–45.1)	100	50.0 (42.0–58.0)
SNF	55 (26.8)	44.0 (35.1–53.2)	100	53.3(45.0–61.5)
PCR	115 (56.1)	92.0 (85.8–96.1)	100	88.9 (80.5–94.5)

N = 205, KK: Kato Katz technique, SNF sodium nitrate flotation.

### Sensitivity

The calculated sensitivities, specificities and NPVs with 95% CI are shown in Table 1. Briefly, the sensitivity of PCR was the highest (92.0%) followed by SNF (44.0%), the quadruple KK (36.0%), day 1 KK (27.2%) and day 2 KK (20.0%) respectively. The specificity of KK, SNF and PCR was assumed 100%.

### Intensity

**Figure 2:**
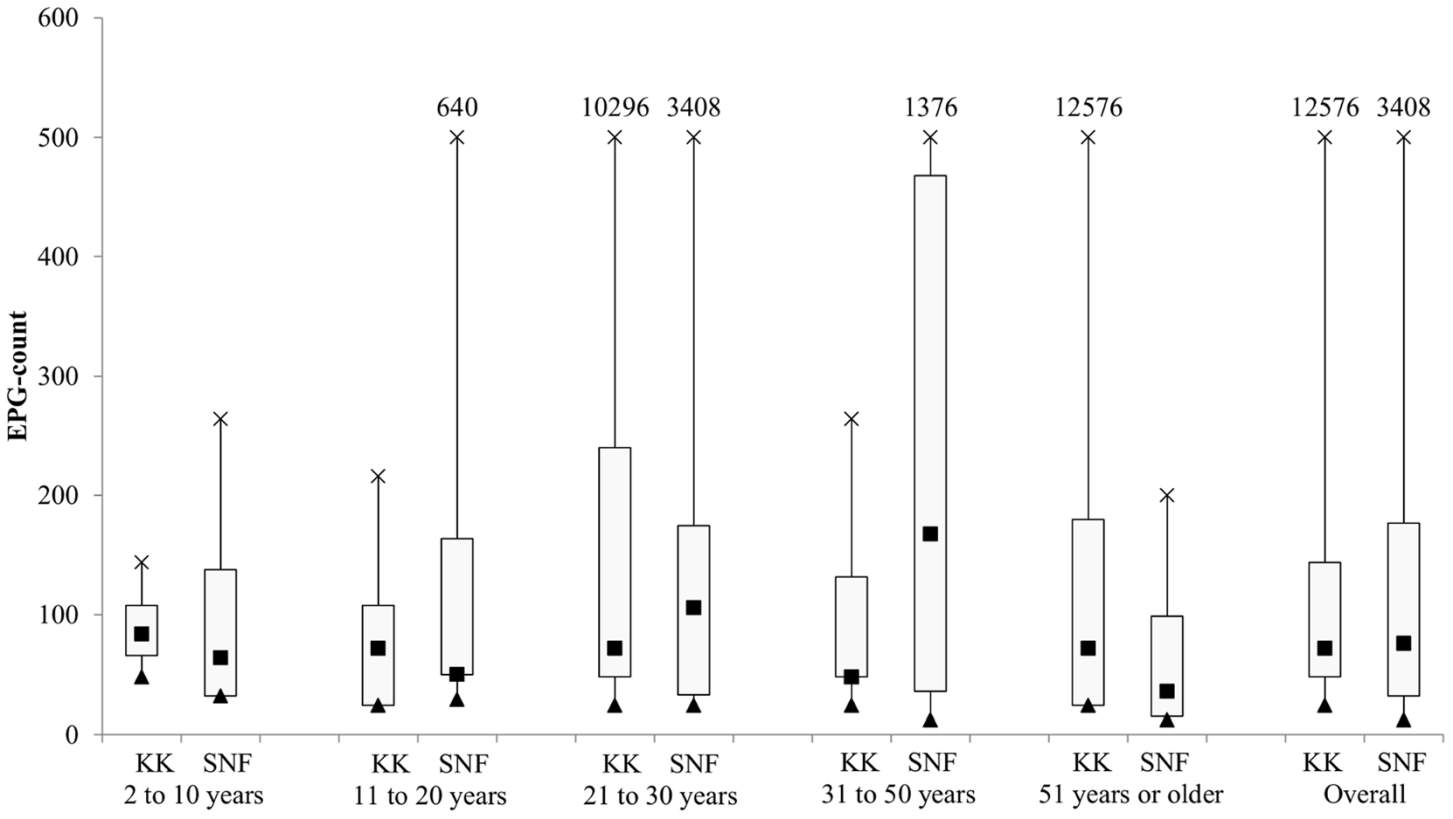
Hookworm infection intensity by age group and examination technique.

Comparison of the median intensity of hookworm infection by age group is shown in Fig. 2. The overall epg count from the quadruple KK was higher than those measured using SNF. However, the epg measured using SNF were higher than quadruple KK in two out of five age groups (21–30 years, 31–50 years). Therefore, there was no significant difference in epg between the two methods. We compared the median epg of the SNF between the samples found only positive by SNF (median epg: 160) to the samples that were analyzed by SNF and KK (median epg: 448). Using the Mann-Withney U test, there was no significant difference in epg values (*P* = 0.121).

**Figure 3:**
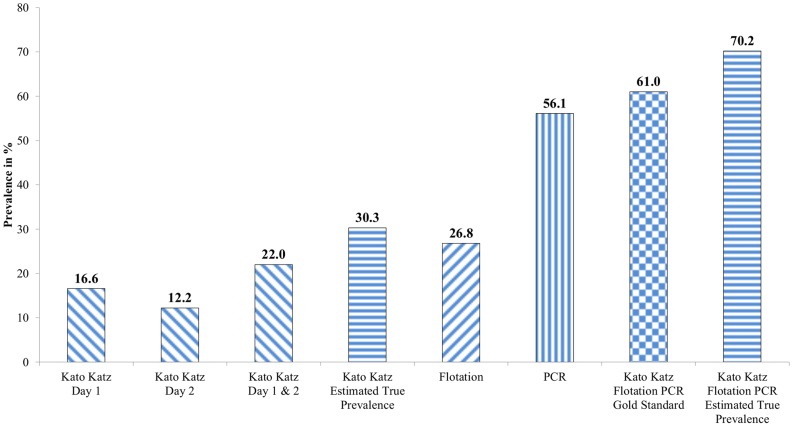
Prevalence (%) of hookworm infection established by each detection technique and estimated true prevalence among 205 humans in Cambodia.

The estimated “true” prevalence for hookworm infection based on the quadruple KK and gold standard were 30.3% and 70.2%, respectively which is an increase of 8.3% from our observed prevalence of 22.0% for quadruple KK and increase of 9.2% from our observed prevalence of 61.0% for gold standard (Fig. 3).

## Discussion

In the present study, three diagnostic techniques (KK, SNF and PCR) were assessed for the qualitative and two techniques (KK and SNF) for the quantitative detection of hookworm eggs in fecal samples from humans in Cambodia. Direct comparison of the three diagnostic techniques showed that the PCR assay had a superior sensitivity compared to the SNF, the single, duplicate and quadruplicate KK techniques. The KK when performed in duplicate with stool samples collected over two consecutive days provided a higher sensitivity (36.0%) for diagnosing hookworm infection when compared to one day KK (day 1 or day 2) alone (27.2% and 20.0%). The ten individuals that were positive by KK and negative by SNF were also found negative on PCR. Therefore it is likely that these 10 positives were false positives on the quadruplicate KK, which is yet another disadvantage of this diagnostic approach. The field of view is poor compared to the SNF and fecal artifacts can be mistaken as helminth eggs. The PCR results are likely explained by two factors: (i) false negatives - the inability to amplify these samples could be associated with failure to remove PCR inhibitors in human stool following DNA extraction [22], (ii) false-positive coproscopy results, i.e. that *Trichostrongylus* eggs detected in stool were misidentified as hookworm eggs. *Trichostrongylus columbricformis* which is present in humans in neighboring countries such as Lao People's Democratic Republic [23] and Thailand [24] produce eggs very similar to hookworms. Although there are no published reports of human infection with this species in Cambodia, *T. columbricformis* infection in humans cannot be disregarded because molecular identification of other strongylid nematodes was not attempted in this study.

The poorer sensitivity of the KK may be directly related to the significantly smaller amount of filtered feces examined (41.7 mg) compared to that of the SNF (∼250 mg). The addition of a washing step in the SNF procedure coupled with flotation provides a relatively ‘clearer’ and debris-free view of the hookworm eggs, thus making microscopic screening and quantification more accurate and time efficient. For a skilled parasitologist, a single SNF would take a maximum of 30 minutes to perform, including quantification of hookworm eggs. Time is thus a significant advantage for the SNF, both in terms of sampling logistics (single versus two stool samples) and preparation and examination of a single instead of duplicate slides. The sensitivity of the KK is further compromised by day-to-day and intra-specimen variation of helminth egg output [13], [25], problems related to delay from time of defecation to collection in the field and processing in the laboratories. Unlike the KK, the SNF has the advantage of indefinite formalin-based fixation at room temperature prior to examination. Rapid over-clearing of hookworm eggs by the KK may also lead to false negatives and/or an under-estimation of hookworm egg intensities [25], [26]. The SNF proved superior to the quadruple KK for the detection of low egg burdens and therefore a better method to monitor the efficacy of anthelmintic treatment programs when worm burdens are expected to be lighter.

The calculation of the “true prevalence” was done for the KK and for the gold standard. It takes into consideration the results of each examination day and estimates the prevalence if unlimited number of samples from the same individual would be examined [21]. This calculation is normally performed only for a specific diagnostic method. Yet, we also performed an estimation calculation for the gold standard, assuming the results of KK day 1, KK day 2, SNF and PCR as four results of the same diagnostic method, performed on four consecutive days.

SNF offers a number of advantages for the detection of hookworm eggs over KK methods. In addition to the superior sensitivity, the SNF did not detect a significant difference in hookworm eggs counts to the KK, a limitation of the FLOTAC technique in which egg counts are consistently reported low by comparison [27], [28]. The SNF technique is simple, quantitative and can be performed using a simple bench-top centrifuge using 10–15 ml disposable centrifuge tubes, surgical gauze, microscope slides and cover slips. Sodium nitrate can be purchased readily from chemical suppliers and if unavailable, the solution can be replaced with saturated salt of equal specific gravity (specific gravity 1.20).

In contrast with SNF, the limitations of the FLOTAC apparatus include its atypical size and the requirements of a large capacity stand-up bucket centrifuge. This requires the procedure be conducted in well-equipped laboratories only [25], usually not present in areas endemic for hookworm infections, including Cambodia. The reusable Fecalyzer device (EVSCO 014008-50, USA) is widely used and available through veterinary suppliers for less than US 1.00 each and may prove an alternative option for conducting SNF in the field or local laboratory. This stand-up fecal flotation device comes with an inbuilt filter and stirrer that in a similar fashion to the mini-FLOTAC, obviates the requirement of a bench-top centrifuge.

In conclusion, our comparison of different techniques suggests that PCR is a highly sensitive technique for the detection of hookworm infection in human parasitological surveys. It offers resource-poor communities a logistically feasible, freely available and cost-effective option to monitor the success of hookworm control programs. The SNF holds promise for the detection of human hookworm and potentially other STH infections and may become an essential tool for patient management, monitoring of helminth control programs and anthelmintic drug efficacy studies in areas with no access to the commercially produced parasitological flotation devices.

## Supporting Information

S1 Checklist STARD checklist.
(DOC)
